# Progress in the study of molecular markers in the prognosis assessment and recurrence patterns of glioblastoma

**DOI:** 10.1080/15384047.2025.2574179

**Published:** 2025-11-07

**Authors:** Yuqing Hu, Xiaoqin Ge, Qianyun Xie, Ruishuang Ma, Qingsong Tao

**Affiliations:** a Department of Tumor Radiotherapy and Chemotherapy, The First Affiliated Hospital of Ningbo University, Ningbo, China; b Department of Medicine, Ningbo University, Ningbo, China

**Keywords:** Glioblastoma, molecular markers, prognosis, recurrence patterns, emerging technologies

## Abstract

Glioblastoma multiforme (GBM), the most invasive primary malignant tumor of the central nervous system, is characterized by an extremely poor prognosis and a high recurrence rate. Its significant molecular heterogeneity challenges precise diagnosis and treatment. Recently, with the rapid development of molecular pathology, the combination of histological and molecular typing has become the mainstream method for GBM diagnosis. Here, we review the impact of classic molecular markers on patient prognosis in GBM, as well as the different values of traditional and novel molecular markers in prognosis assessment. We initially discuss the correlation between molecular markers and recurrence, as well as the research progress of molecular markers in emerging technological fields. Moreover, we propose the challenges currently faced by molecular markers in glioblastoma and discuss future research directions in this field.

## Introduction

Glioblastoma multiforme (GBM) represents the most aggressive primary malignant tumor of the central nervous system, comprising approximately 48.6% of all malignant central nervous system neoplasms.^1^^,^^2^ Despite multimodal therapeutic approaches including maximal surgical resection, radiation therapy, and chemotherapy, patient outcomes remain dismal, with a median overall survival of typically less than 15 months,^3^^,^^4^ and the 5-year survival rate is only 5%–10%.^2^^,^^4^ This poor prognosis is primarily attributed to the highly invasive characteristics and substantial molecular heterogeneity of these tumors. In the current era of precision medicine, conventional histopathological assessment alone proves insufficient for clinical decision-making; consequently, the implementation of molecular markers offers novel approaches for prognostic stratification and recurrence monitoring in GBM patients.

With the widespread application of high-throughput sequencing technology, multiple studies have confirmed that specific molecular markers are significantly associated with the clinical outcome of GBM. The difference in molecular expression leads to survival differences at the individual level; for example, patients with glioblastoma carrying wild-type IDH1/2 have a shorter median survival period compared to other gliomas.^5^ To further improve prediction accuracy, current research trends tend to adopt an integrated analysis strategy involving multiple molecular markers. This involves jointly evaluating the methylation status of the MGMT promoter, TERT promoter mutations, EGFR amplification, and the deletion of CDKN2A/2B to establish a more precise prognostic stratification system.

These research advances not only deepen our comprehension of the molecular heterogeneity of GBM but also lay the theoretical foundation for developing new prognostic assessment systems and targeted treatment strategies. Pediatric glioblastoma differs from adult glioblastoma at the molecular level, which may lead to differences in prognosis.^6^ Therefore, this review seeks to elucidate the impact of molecular markers in adult glioblastoma on patient prognosis and recurrence patterns, as well as the application of emerging technologies in the study of molecular markers.

## The correlation of GBM's classic molecular markers with prognosis

### **IDH mutations**

The IDH mutation status constitutes a fundamental biomarker in the molecular typing of GBM, exerting a profound influence on patient prognosis and therapeutic stratification. IDH1 is a key metabolic enzyme in the Krebs cycle, catalyzing the oxidation and decarboxylation of isocitrate to *α*-ketoglutarate. In GBM, IDH1mut patients have significantly improved survival compared to IDH1wt patients. This biological difference may be related to metabolic reprogramming and abnormal epigenetic regulation induced by mutations. The mutant IDH1 can catalyze the conversion of *α*-ketoglutarate to 2-hydroxyglutarate. Owing to its similar molecular structure, 2-hydroxyglutarate can competitively inhibit *α*-ketoglutarate-dependent enzymes, thereby inhibiting the malignant progression of tumors.^7^ Although there is controversy regarding the impact of 2-hydroxyglutarate on GBM, the different survival outcomes of the two groups suggest that it may be related to different metabolic pathways. Further exploration is needed to understand the differences in the two metabolic patterns. Recent research data indicate that approximately 90% of cases are IDH wild-type (IDHwt), while IDH mutant (IDHm) tumors account for approximately 10% of cases.^8^ In the fifth edition of the “Classification of Central Nervous System Tumors,” IDHm-GBM has been redefined as “IDH-mutant astrocytoma, CNS WHO grade 4,” and GBM diagnosis is only applicable to IDHwt tumors, further establishing the central role of the IDH status in prognosis assessment.^9^^,^^10^

Notably, IDHwt-GBM also exhibits significant tumor invasiveness and complex molecular heterogeneity, responding poorly to conventional treatments such as surgery and chemoradiotherapy. Its prognosis is influenced by multiple molecular features, including EGFR amplification, TERT promoter mutations, and MGMT promoter methylation status.^11^

### **MGMT promoter methylation**

O6-methylguanine-DNA methyltransferase (MGMT) promoter methylation has been confirmed to be a key determinant of prognosis in GBM and a potential predictive factor for the temozolomide (TMZ) response. As a ubiquitous DNA repair enzyme in cells, MGMT can protect chromosomes from damage caused by alkylating agents, and its expression is controlled by DNA methylation in the promoter region. High methylation leads to reduced gene transcription and MGMT protein expression, thereby impairing the repair of DNA damage caused by alkylating agents, which results in improved treatment responses and longer survival periods. In particular, patients with MGMT promoter methylation who receive combined radiotherapy and chemotherapy with alkylating agents can achieve more significant therapeutic benefits compared to unmethylated patients.^12^ For elderly or frail patients, the status of the MGMT promoter should be considered more carefully when choosing a treatment plan: methylated patients may benefit from radiotherapy combined with TMZ. In contrast, the treatment plan for unmethylated patients may need to be adjusted to avoid ineffective chemotherapy and avoid over-treatment toxicity.^13^

Given the importance of MGMT in the TMZ resistance mechanism, various methods have been used to increase the sensitivity of TMZ. In one study, the MGMT promoter was targeted for genome editing using the CRISPR off-target tool, leading to MGMT promoter methylation. This approach overcomes TMZ resistance in known glioblastoma cell lines and induced cell death through TMZ. This study demonstrated that targeted RNA-guided methylation of the MGMT promoter can effectively overcome chemoresistance and enhance the cytotoxicity of TMZ.^14^ While MGMT promoter methylation editing technology has not yet reached the clinical stage, it provides a foundation for experimental alkylating agent treatment strategies.

Interestingly, only a small portion of recurrent GBM patients exhibit dynamic changes in MGMT promoter methylation status during the progression of the disease.^15^ This dynamic evolution may be related to clonal selection under treatment pressure, indirectly suggesting the need for dynamic monitoring of molecular marker changes throughout the management of GBM to guide clinical treatment decisions. The clinical therapeutic value of the MGMT promoter methylation status in neuro-oncology is widely recognized; however, multiple studies have identified inconsistencies between the methylation levels and clinical prognosis. MGMT promoter methylation shows varying degrees of survival improvement across different age groups, subtypes, and treatment regimens.^12^^,^^16^

### **EGFR and other key molecular markers**

In the current classification of GBM, key molecular markers such as EGFR amplification, TERT promoter mutations, and chromosomal alterations +7/-10 are used as diagnostic criteria. Improving the accuracy of GBM diagnosis through molecular diagnosis is crucial for selecting appropriate personalized treatment strategies. The tumor heterogeneity of GBM is driven by the alteration of many interrelated signaling pathways, highlighting the importance of targeting single molecular markers for effective treatment. The epidermal growth factor receptor (EGFR) plays a crucial role in regulating cell division, migration, adhesion, differentiation, and apoptosis and can trigger multiple downstream signaling pathways, particularly the PI3K-AKT-mTOR pathway. These pathways are involved in cell proliferation, survival, and tumor invasion, thereby promoting disease progression.^17^ Therefore, EGFR is often associated with poor prognosis in GBM. However, a study revealed that EGFR amplification is associated with different survival outcomes depending on the levels of different ligands. According to TCGA data analysis, in GBM with EGFR amplification, low levels of EGFR ligands are associated with poorer prognosis, while high levels of EGFR ligands are associated with improved prognosis. This may be related to the upregulation of EGFR ligands suppressing the DOCK−7 Rho GTP pathway via BIN3, resulting in smaller in situ tumors, less invasive behavior, and higher survival rates.^18^ Notably, EGFR and TP53 mutations are almost mutually exclusive. When both processes occur simultaneously, they may lead to poorer clinical outcomes in GBM patients, which may be related to the related cell cycle dysregulation caused by the simultaneous dysregulation of the EGFR and p53 pathways.^19^^,^^20^

The infinite proliferative ability of tumors depends not only on EGFR regulation but also on the telomere maintenance mechanism. Multiple studies have reported that the telomerase reverse transcriptase promoter (TERTp) is the most common clonally activating mutation in glioblastoma multiforme (GBM), maintaining telomere length through the overexpression of telomerase reverse transcriptase (TERT) to achieve genomic stability in tumor cells.^21^ Despite this, the potential prognostic role of TERTp mutations remains controversial. In a single-center study, TERTp was identified as an independent prognostic factor for improved spastic progression; however, its mutation status did not impact the clinical outcome of GBM.^22^ Simon et al. noted that TERTp mutations do not have a prognostic impact on patients who undergo complete resection and receive temozolomide chemotherapy, but they are prognostically relevant in patients with residual tumors who have not received temozolomide chemotherapy. Surgery and TMZ chemotherapy are effective against tumor cells caused by TERTp mutations compared to surgery and radiotherapy alone, which indicates that tumors with TERTp mutations may require more aggressive surgical and chemotherapy strategies. However, the specific mechanisms remain to be elucidated.^23^ This finding also indirectly suggests that TERTp mutations may have the potential to serve as therapeutic stratification biomarkers.

The coexistence of the 7th chromosome gain (including the EGFR gene) and the 10th chromosome deletion (including PTEN and other tumor suppressor genes) (+7/-10) constitutes the basic molecular characteristic of GBM pathogenesis.^24^ This chromosomal imbalance promotes tumor proliferation and invasion through gene dose effect and is associated with the highest histological grade of IDHwt-GBM.^25^ The presence of chromosome +7/-10 is significantly associated with a shortened survival period in patients, possibly driving tumor progression through compensatory mechanisms, such as the overexpression of genes on chromosome 7, which offsets the adverse effects of the deletion on chromosome 10.^24^^,^^26^ Notably, in low-grade gliomas, the combination of chromosome +7/-10 with TERT mutation or EGFR amplification is considered an independent molecular marker of the worst clinical outcome.^27^

**Figure 1.**
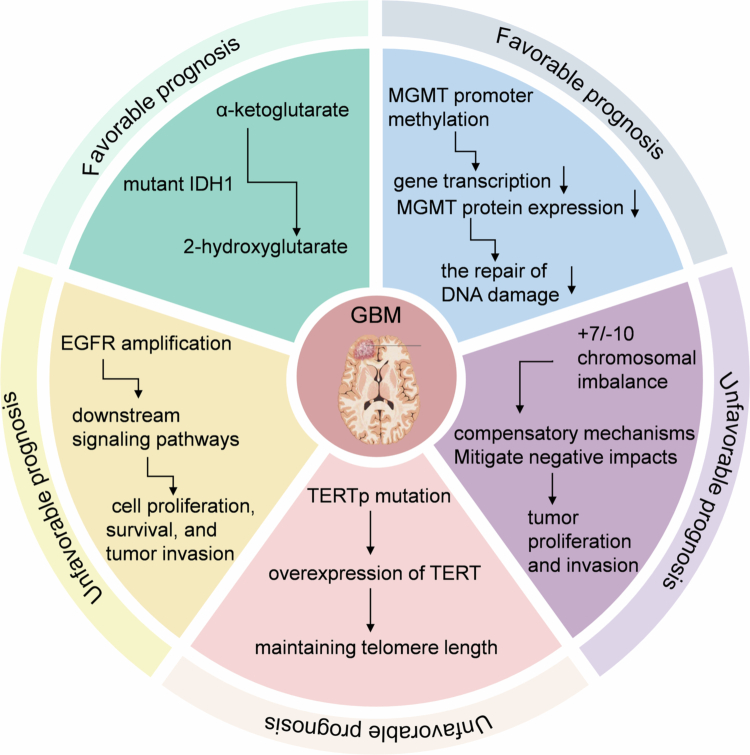
Correlation of classic molecular markers in GBM with patient prognosis. 1. IDH mutations convert *α*-ketoglutarate to 2-hydroxyglutarate, competitively inhibiting *α*-ketoglutarate-dependent enzymes and inhibiting the malignant progression of tumors. 2. MGMT promoter methylation leads to a reduction in gene transcription and MGMT protein expression, thereby impairing the repair of DNA damage caused by alkylating agents, resulting in better survival prognosis. 3. EGFR participates in cell proliferation, survival, and tumor invasion by regulating multiple downstream signaling pathways, promoting disease progression. 4. TERTp mutations lead to the overexpression of TERT, maintaining telomere length, achieving genomic stability in tumor cells, and resulting in a poor prognosis. 5. Chromosomes +7/-10 compensate for the negative effects of chromosomal imbalance, promoting tumor proliferation and invasion.

Given that EGFR amplification, TERT mutations, and chromosome +7/-10 are often observed simultaneously, these three factors have been included in the WHO 2021 classification of central nervous system tumors as diagnostic criteria for IDHwt-GBM.^28^^,^^29^ The simultaneous presence of these molecular markers suggests that the synergistic action of multiple pathways may accelerate a highly invasive phenotype. Further analysis indicates that the synergistic action of EGFR-driven proliferation, TERT-mediated immortalization, and chromosomal imbalance leads to genomic instability.^30^^,^^31^ However, a minority of IDHwt-GBM patients lack these typical markers and may maintain a malignant phenotype through other alternative molecular mechanisms, such as PDGFRA amplification and TP53/NF1 mutations. The clinical behavior and prognosis of these patients need to be further analyzed in conjunction with molecular subtypes.^32^^,^^33^ (The classic molecular markers and prognostic correlation of glioblastoma are shown in Figure 1).

## The application of traditional and emerging molecular biomarkers in the prognostic assessment of GBM

### **Prognostic value of traditional molecular biomarkers**

In the prognostic stratification system of GBM, molecular markers play crucial predictive role. The IDH mutation status is an important diagnostic indicator for molecular typing, through which it alters tumor metabolic pathways (such as reducing *α*-ketoglutarate synthesis) to slow disease progression. Therefore, patients with IDH-mutant astrocytoma, CNS WHO grade 4, exhibit longer survival and better treatment responses compared to patients with IDH-wildtype glioblastoma.^5^^,^^7^ In terms of predicting treatment response, MGMT promoter methylation has significant clinical importance; GBM patients with methylated MGMT show greater sensitivity to TMZ chemotherapy, thereby achieving greater survival benefits.^34^

In addition to the well-known classic molecular markers, CDKN2A/B homozygous deletion is also an important prognostic factor. CDKN2A/B homozygous deletion promotes malignant tumor behavior by causing cell cycle disorder and increasing cell proliferation. Some reports indicate that in IDH-mutant astrocytoma, homozygous deletion of CDKN2A/B is a significant molecular marker for malignant transformation and is strongly associated with poor clinical outcomes.^35^^,^^36^ However, the independent clinical prognostic significance of CDKN2A/B homozygous deletion in IDH-wildtype glioblastoma may be important only in tumors with unmethylated MGMT promoters, where overall survival (OS) is slightly lower compared to tumors without CDKN2A/B homozygous deletion, but this difference is statistically significant (median OS of 14.7 months and 16.9 months), which is not observed in MGMT methylated tumors.^37^ The functional correlation between CDKN2A/B homozygous deletion and invasive tumor behavior in gliomas may result from direct interference with IDH-regulated tumor biology.

### **The impact of emerging molecular markers on survival outcomes**

In recent years, research has revealed that, in addition to traditional molecular markers, emerging molecular markers have gradually demonstrated potential prognostic value. The p16^INK4A^ protein encoded by the CDKN2A gene, a cyclin-dependent kinase (CDK) inhibitor, can induce cell cycle arrest, ultimately leading to cellular senescence. An investigation of the impact of p16^INK4A^ expression in GBM revealed that GBM with higher p16^INK4A^ expression is associated with senescence phenotypes and greater infiltration of immune cells compared to GBM with lower p16^INK4A^ expression. Subsequent in vitro studies have shown that CCL13 released from senescence-inducing GBM cells overexpressing p16^INK4A^ can recruit T cells to the tumor tissue, which may help construct a more immunogenic tumor microenvironment, thereby improving the prognosis of GBM patients.^38^

XRN2, as a 5'-3' ribonuclease, shows significantly upregulated expression levels in GBM tissue. This molecule enhances cellular migration capability by regulating the expression of genes related to tumor invasion. Overexpression of XRN2 can lead to poorer overall survival rates and increased tumor invasiveness in patients.^39^

The downstream αB-crystallin encoded by the CRYAB gene can inhibit the process of cell apoptosis and promote the proliferation, migration, and invasion of tumor cells by regulating proteins involved in the cell cycle. High expression of CRYAB may promote the differentiation of tumor cells or inhibit the immune microenvironment, thereby reducing the sensitivity of GBM to treatment.^40^ As a predictive indicator of poor prognosis, CRYAB targeting in the future may become a promising therapeutic strategy for glioblastoma.

In terms of regulating the tumor immune microenvironment, the discovery of the ALDOC-PPAR-*γ* axis has opened up new avenues for targeted therapy, with PPAR-*γ* agonists exerting anti-tumor effects by regulating metabolic and inflammatory response pathways.^41^ High expression of APOBEC3C (A3C) is associated with an immunosuppressive state in the tumor microenvironment, and this molecule accelerates tumor progression by inducing genomic instability. Its expression level is significantly correlated with a poor patient prognosis.^42^ These molecular markers influence the prognosis of GBM by regulating key pathways, including tumor metabolism, invasion, and immune evasion, thereby providing a molecular basis for the development of personalized treatment strategies. (The summary of the molecular markers for glioblastoma is shown in Tables 1 and 2).

**Table 1.** Comparison of traditional and emerging molecular markers in glioblastoma.

Category	Biomarkers	Main features and mechanisms	Prognostic value	Clinical application method
**Traditional molecular markers**	IDH mutation	It is the foundation of molecular typing. IDH mutations alter tumor metabolism pathways (such as reducing *α*-ketoglutarate synthesis), delaying disease progression.	**Favorable prognostic indicators.** Patients with IDH-mutant astrocytoma and CNS WHO grade 4 disease have significantly longer survival periods and better treatment responses than do those with IDH-wildtype glioblastoma.	**1. Diagnosis and typing:** To confirm GBM and distinguish between IDH wild-type glioblastoma, CNS WHO grade 4, and IDH-mutant astrocytoma, CNS WHO grade 4, which is a core indicator in the WHO grading system. **2. Prognostic stratification:** Provides initial prognostic information for patients, serving as an important basis for clinical decision-making and patient consultation. **3. Treatment exploration:** Inhibitors targeting IDH mutant enzymes (such as Vorasidenib) are currently in clinical trials and are expected to become targeted treatment strategies.
	MGMT promoter methylation	MGMT promoter methylation leads to the silencing of the DNA repair enzyme MGMT, making tumor cells unable to repair DNA damage caused by TMZ.	**Favorable prognostic indicators.** Patients who are MGMT methylation positive are more sensitive to TMZ chemotherapy and have more significant survival benefits.	**1. Treatment prognosis:** A Core Clinical Application. The ability to predict a patient's sensitivity to TMZ chemotherapy is a key reference for formulating first-line chemotherapy regimens. MGMT methylation-positive patients are strongly recommended to receive TMZ treatment. **2. Prognosis assessment:** Combines other molecular markers to comprehensively judge patient prognosis.
	EGFR amplification	EGFR triggers multiple downstream signaling pathways (such as the PI3K-AKT-mTOR pathway), which are involved in cell proliferation, survival, and tumor invasion. The level of its ligand can affect tumor invasiveness by inhibiting the DOCK−7 Rho GTP pathway.	EGFR amplification is usually associated with poor prognosis, but high expression of its ligands may improve patient prognosis. It is mutually exclusive with TP53 mutations; if both are present, it indicates **a very poor prognosis**.	**1. Diagnostic criteria:** IDH wild-type GBM is one of the core indicators for diagnosing IDH wild-type GBM according to the WHO. **2. Prognostic assessment:** Patients need to be comprehensively judged by combining their ligand expression levels and TP53 status to identify subgroups of patients with extremely poor prognoses. **3. Treatment target:** TERT is an important candidate molecule for targeted therapy, although targeted therapies against EGFR are available.
	TERT promoter mutations	TERT promoter mutations are the most common clonally activating mutations, maintaining telomere length through TERT overexpression and achieving immortalization of tumor cells.	**There is controversy.** Its prognostic value may depend on the treatment approach. Patients who receive TMZ chemotherapy and complete resection may not have an independent prognostic impact, but those who do not have complete resection or who have not received chemotherapy may have a **poor prognosis**.	**1. Diagnostic criteria:** IDH wild-type GBM is one of the core indicators for diagnosing IDH wild-type GBM according to the WHO. **2. Treatment stratification: It has the potential to serve as a treatment stratification biomarker.** Studies have shown that tumors harboring this mutation may require more aggressive surgical resection and TMZ chemotherapy strategies.
	Chromosome +7/-10	Chromosome 7 gain (containing EGFR) and chromosome 10 deletion (containing PTEN) coexist, promoting proliferation and invasion through a gene dosage effect.	**Clear adverse prognostic indicators.** It is associated with significantly shortened patient survival. In low-grade gliomas, the combination of TERTp mutation or EGFR amplification indicates **the worst clinical outcome**.	**1. Diagnostic criteria:** One of the core indicators for diagnosing IDH wild-type GBM according to the WHO. **2. Prognostic assessment:** A strong poor prognostic factor is used to identify the most aggressive tumor types.
	CDKN2A/B	CDKN2A/B homozygous deletion leads to the loss of cell cycle regulatory proteins (such as p^16INK4A^), causing cell cycle dysregulation and increased proliferation.	**Poor prognostic indicators.** IDH-mutant astrocytoma, with a CNS WHO grade of 4, is strongly associated with malignant transformation and poor outcomes. GBM patients with unmethylated MGMT promoters also have a poorer prognosis.	**1. Risk stratification:** This method is mainly used for refined prognostic stratification of IDH-mutant astrocytoma. Even with IDH mutation, if accompanied by CDKN2A/B homozygous deletion, the prognosis risk level significantly increases, which may affect the intensity of treatment strategies (such as more aggressive treatment or enrollment in clinical trials). GBM is mainly used for prognostic stratification of patients with an unmethylated MGMT promoter. **2. Auxiliary diagnosis:** Supports the diagnosis of high-grade gliomas.
**Emerging molecular biomarkers**	p^16INK4A^ (encoded by the CDKN2A gene)	p^16INK4A^ induces cell cycle arrest and cellular senescence. High expression of p^16INK4A^ can recruit T cells (through CCL13) and shape a more immunogenic tumor microenvironment.	**Favorable prognostic indicators.** High expression of p^16INK4A^ is associated with increased immune cell infiltration and better prognosis.	**1. Prognostic assessment:** p^16INK4A^ serves as a potential favorable prognostic marker, particularly in analyses related to the immune microenvironment. **2. Treatment development:** This study provides new insights for immunotherapy strategies, such as developing combination therapies that induce senescence in tumor cells followed by the activation of antitumor immunity.
	XRN2	XRN2, as a 5' to 3' ribonuclease, enhances the migratory and invasive abilities of tumor cells when upregulated.	**Poor prognostic indicators.** XRN2 overexpression leads to shorter overall survival and increased tumor invasiveness.	**1. Prognosis assessment:** Identify high-risk patients with a highly invasive phenotype. **2. Treatment Targets:** Inhibiting XRN2 may serve as a potential treatment target to reduce tumor invasion and metastatic ability.
	CRYAB (αB-crystallin)	CRYAB inhibits cell apoptosis, regulates cell cycle proteins, and promotes proliferation, migration, and invasion. It may also inhibit the immune microenvironment.	**Poor prognosis indicators.** High expression of CRYAB reduces treatment sensitivity, indicating a poor prognosis.	**1. Prognosis assessment:** A predictive indicator of poor prognosis. **2. Treatment Target: A highly promising treatment target.** Future development of CRYAB inhibitors may lead to a new strategy to overcome treatment resistance and improve patient prognosis.
	APOBEC3C (A3C)	APOBEC3C (A3C) induces genomic instability, accelerating tumor progression; it is associated with immune suppression in the tumor microenvironment.	**Poor prognosis indicators.** High expression of APOBEC3C (A3C) is significantly associated with poor prognosis.	**1. Prognosis assessment:** Identify high-risk patients with high levels of genomic instability and immunosuppression. **2. Treatment targets:** Not only can they serve as treatment targets, but their expression levels in the future may also be used to predict a patient's response to **immune checkpoint inhibitors**.

**Table 2.** Summary of molecular markers for glioblastoma.

Biomarkers	Prognostic effect strength	Support verification level	Association with recurrence/progression	Summary of key clinical significance
**IDH mutation**	**Strong** (favorable factors)	**Level 1** (core diagnostic criteria for CNS WHO classification)	**Clarify relevant** (IDH wild-type is more prone to recurrence)	It is **the most decisive prognostic factor for GBM**. IDH-mutant astrocytoma (now termed IDH-mutant astrocytoma, CNS WHO grade 4) has a significantly better prognosis than IDH wild-type astrocytoma (classic GBM).
**MGMT promoter methylation**	**Strong** (favorable factors)	**Level 1** (substantially confirmed by clinical studies, basic consensus)	**Clarify relevant** (related to treatment resistance and recurrence)	It is **a powerful prognostic and predictive marker**. Methylation status predicts sensitivity to TMZ chemotherapy and longer survival. It is a key basis for treatment decisions in elderly/frail patients.
**Chromosomes +7/-10**	**Medium-Strong** (Adverse Factors)	**Level 1** (One of the diagnostic criteria for IDH wild-type GBM)	**Clarify relevant** (Promote tumor invasion and progression)	It is a diagnostic molecule for IDH wild-type GBM, associated with strong tumor proliferation, invasion, and **significantly shortened survival**.
**EGFR amplification**	**Medium** (Adverse Factors, but subject to background adjustment)	**Level 1** (One of the diagnostic criteria for IDH wild-type GBM)	**Clarify relevant** (Promote proliferation and invasion pathways)	It is a classic oncogenic driver, typically associated with poor prognosis. However, its impact may be complex due to factors such as ligand expression levels and TP53 comutation status.
**TERT promoter mutation**	**Medium** (Adverse factors, prognosis varies with different treatment backgrounds)	**Level 1** (one of the diagnostic criteria for IDH wild-type GBM)	**Possibly relevant** (related to treatment resistance)	It is one of the essential diagnostic markers, **but its independent prognostic value is controversial**. According to the fourth edition of the “WHO Classification of Tumors of the Central Nervous System,” TERT mutation is a favorable factor in IDH-mutant glioblastoma multiforme, while TERT mutation is an adverse factor in IDH-wild-type glioblastoma multiforme. However, in the fifth edition of the “WHO Classification of Tumors of the Central Nervous System,” IDH-mutant glioblastoma multiforme was redefined as IDH-mutant astrocytoma, and in IDH-wildtype GBM, TERT mutation may not be a simple prognostic factor; its significance depends on the treatment strategy (such as the response to TMZ), and more aggressive treatment is needed.
**CDKN2A/B homozygous deletion**	**Medium**(Adverse Factors)	**Level 2** (Previously, the absence of necrosis and microvessel proliferation in histology was one of the diagnostic criteria for IDH-mutant glioblastoma, and now it is one of the diagnostic criteria for IDH-mutant astrocytoma.)	**Possibly relevant** (Promote malignant progression and transformation)	In IDH-mutant astrocytoma, it is a strong risk factor indicating an increased malignancy and extremely poor prognosis. In IDH-wildtype GBM, its negative impact may be limited to the MGMT nonmethylated subgroup.
**High expression of XRN2**	**Medium** (Adverse Factors)	**Level 3** (Emerging evidence, limited research)	**Possibly relevant** (Enhanced assault capability)	A ribonuclease exosome whose expression is upregulated in GBM. **Overexpression is significantly associated with poorer overall survival and enhanced tumor invasiveness**, enhancing cell migration ability by regulating invasion-related genes.
**CRYAB (αB-crystallin)**	**Weak** (Adverse Factors)	**Level 3** (Emerging evidence, limited research)	**Insufficient evidence** (promotion of proliferation, invasion, and treatment resistance)	can inhibit apoptosis, regulate the cell cycle, and promote the proliferation, migration, and invasion of tumor cells. High expression can reduce treatment sensitivity and is **a predictive indicator of poor prognosis**, as well as a potential therapeutic target.
**APOBEC3C (A3C)**	**Weak** (Adverse Factors)	**Level 3** (Emerging evidence, limited research)	**Insufficient evidence** (induced genomic instability and accelerated progression)	is associated with the immunosuppressive state of the tumor microenvironment. By inducing genomic instability, it accelerates tumor progression, and its expression level is significantly associated with **poor prognosis** in patients.
**p** ^**16INK4A**^ **(encoded by the CDKN2A gene)**	**Weak** (Favorable Factors)	**Level 3** (emerging evidence, limited research)	**Insufficient evidence** (delay progress)	is **the functional protein product of the CDKN2A gene**. High expression can induce cell cycle arrest and senescence. New evidence suggests that senescent GBM cells may recruit T cells by releasing chemokines such as CCL13, shaping an immunogenic tumor microenvironment and **potentially improving patient prognosis**.

## The relationship between GBM recurrence patterns and molecular markers

### **Spatial features of GBM recurrence**

The recurrent GBM exhibits significant anatomical preference characteristics. Studies have shown that the peritumoral brain zone (PBZ) constitutes the primary pathological basis for tumor recurrence, with clinical data indicating that approximately 90% of recurrence events occur in this region. This special anatomical area not only contains residual infiltrating tumor cell subpopulations but also has a unique immunosuppressive microenvironment and abnormal vascular networks that collectively promote the recurrence process.^43^^,^^44^ Notably, GBM located in the vicinity of the lateral ventricles exhibits more aggressive clinical features, including significantly shortened survival periods and increased distant recurrence rates, which may be related to the tumor cell dissemination mechanism mediated by the cerebrospinal fluid circulation system, particularly the key roles played by epidermal growth factor (EGF) and vascular endothelial growth factor (VEGF).^45^ From a surgical perspective, the cavity margin of the resection is another hotspot for recurrence, with over 80% of recurrences occurring here, indicating that residual tumor cells post-surgery can rapidly proliferate and develop treatment resistance under the stimulation of local hypoxia and enriched inflammatory factors.^43^ These findings provide important pathological and physiological bases for determining the scope of surgical resection and developing local targeted therapies.

### **The correlation between the spatial and molecular characteristics of recurrent GBM**

The invasive margin of GBM shows significant molecular differences compared to the core region. The 5ALA-positive cell population (5ALA+) at the invasive margin exhibits a typical mesenchymal phenotype (upregulated VIM and FN1) and abnormal activation of pro-inflammatory cytokine signaling pathways, particularly the IL-6/JAK/STAT3 pathway. This area forms a complex intercellular interaction network with tumor-associated macrophages (M2 TAMs), collectively shaping a local microenvironment that is conducive to tumor invasion and immune suppression, which may be an important mechanism for early recurrence.^46^ In contrast, the tumor core region is enriched with genes related to cell proliferation (such as MKI67), while cells in the invasive margin express high levels of migration-related molecules (such as CD44 and the MMP family) and stress response genes (such as HSPB1/CRYAB). Additionally, CRYAB and CD44 are highly co-expressed in aggressive GBM, synergistically regulating cytoskeletal remodeling and anti-apoptotic signaling pathways, which significantly enhances the tumor's recurrence after surgery.^47^ This spatial heterogeneity not only reveals the molecular basis of GBM invasiveness but also provides potential intervention strategies targeting the tumor margin microenvironment to suppress recurrence.

### **Potential molecular markers for predicting recurrence patterns**

Despite comprehensive treatment, recurrence is the norm for GBM. According to the spatial relationship between the recurrent tumor and the target area of radiotherapy, recurrence patterns are classified as central recurrence, intra-field recurrence, marginal recurrence, and extra-field recurrence. Different recurrence patterns are associated with various factors, and it is undeniable that molecular genetics plays a decisive role in GBM recurrence patterns.

Scholars have used targeted exome sequencing to screen patients with both initial and recurrent IDHwt-GBM in databases. By evaluating the continuity of recurrent tumors based on MRI at initial diagnosis and recurrence, they concluded that a minority of IDHwt-GBM patients experience discontinuous recurrence.^48^ Thus, it can be inferred that IDHwt-GBM is closely related to local recurrence.

MGMT promoter methylation is considered a good prognostic factor in GBM, but it has the opposite effect on recurrence patterns. A meta-analysis including the MGMT methylation status showed that MGMT promoter methylation is significantly associated with non-local recurrence (OR: 1.55, 95% CI: 1.09−2.20).^49^ However, the specific impact of MGMT promoter methylation on recurrence patterns remains controversial, and the current pathophysiological mechanisms cannot explain the occurrence of distant recurrence.

However, a prospective study revealed the relationship between hotspot mutations in the extracellular domain of EGFR and distant recurrence. The study divided GBM patients who received chemoradiotherapy combined with TMZ into the EGFR^A289^ wild-type and mutant groups. A comparison of the overlap scores of the initial irradiated tumor volume (Vinit) and the recurrent volume (Vr) revealed that the mutant EGFR^A289^ is more prone to edge recurrence, which may be related to the activation of the MAPK pathway, thereby promoting tumor proliferation and invasion.^50^ However, this study did not further explore the molecular characteristics of recurrent tumors. There may be changes in the molecular characteristics of recurrent tumors after treatment.

The 5'-3' ribonuclease XRN2 mediates the dissemination of GBM, but the specific mechanism remains unclear. Some scholars believe that this may be related to XRN2's ability to promote cell movement and migration. In cell experiments, the movement ability of cells lacking XRN2 was significantly reduced.^39^ Therefore, XRN2 may be involved in the distant recurrence of GBM.

The discovery of these markers not only facilitates the early identification of high-risk patients but also provides a theoretical basis for developing personalized treatment strategies that target recurrence patterns.

## Emerging technologies and molecular biomarkers

### **Circulating tumor DNA (ctDNA) in liquid biopsy**

Liquid biopsy technology, which analyzes tumor-derived molecules such as circulating tumor DNA (ctDNA) in the body fluids of patients with central nervous system tumors, including GBM, has become a promising minimally invasive diagnostic approach. In terms of early diagnosis and recurrence monitoring, ctDNA in the blood can be used to sensitively detect minimal residual disease (MRD) post-surgery and provide early warning of disease recurrence. The feasibility of ctDNA detection has been verified in pediatric brain tumors, indicating its potential for transformation in GBM.^51^ Dynamically monitoring of ctDNA levels can serve as a molecular surrogate for evaluating the efficacy of chemotherapy or targeted therapy. Studies have shown that a decrease in ctDNA mutation frequency during treatment is significantly associated with clinical remission.^52^ Additionally, ctDNA can assist in molecular typing and treatment decision-making. Through cerebrospinal fluid (CSF) ctDNA, IDH mutations and MGMT promoter methylation, key molecular markers can be detected,^53^ aiding in the formulation of personalized treatment plans. These advancements demonstrate the diagnostic potential of ctDNA as a liquid biopsy tool in the precise diagnosis and treatment of GBM.

However, liquid biopsy based on ctDNA still faces technical bottlenecks in GBM, resulting in low clinical application value in glioblastoma. The blood–brain barrier significantly reduces the concentration of ctDNA in the blood, resulting in insufficient sensitivity in plasma-based liquid biopsies.^54^^,^^55^ Comparative studies have shown that ctDNA detection rates are relatively high in CSF because of direct contact with the tumor microenvironment.^56^ The lack of GBM-specific biomarkers limits the precision of detection; current research primarily focuses on targeted analysis of known mutations (such as H3F3A K28M or BRAF V600E).^52^^,^^53^ The difficulty in obtaining samples further restricts the practical clinical application: although CSF liquid biopsy has relatively high sensitivity, lumbar puncture carries risks associated with invasive procedures and infection.^57^^,^^58^

These challenges urgently require technological innovation and the development of new biomarkers to overcome bottlenecks, such as optimizing the sensitivity issues in CFS ctDNA detection by combining ultra-sensitive ddPCR and methylation-specific sequencing techniques. Additionally, integrating exosome protein biomarker detection is expected to enhance dynamic monitoring effects.

### **Multi-omics analysis in molecular marker research**

In recent years, multi-omics integrative analysis methods have become important tools for studying the molecular heterogeneity of GBM and discovering new molecular biomarkers. As GBM is a highly heterogeneous malignant tumor, the complex molecular characteristics of GBM are considered the primary factors contributing to clinical treatment failure. By systematically integrating genomic, transcriptomic, proteomic, and metabolomic data, researchers can comprehensively analyze molecular differences among tumor cell subpopulations. For example, spatial multi-omics technologies, which combine single-cell and spatial molecular features, have revealed interactions between malignant cells and stromal cells within the glioblastoma tumor microenvironment (TME). It has been confirmed that metabolic reprogramming (especially lipid metabolic disorders) in tumor-associated myeloid cells (TAMCs) in hypoxic microenvironments is an important mechanism for inducing an immunosuppressive state.^59^^,^^60^ In a multi-omics analysis of 68 cases of high-grade gliomas from multiple regions, researchers proposed that tumors located in MRI non-enhancing invasive areas promote tumor malignancy through metabolic adaptation mechanisms and epigenetic regulation.^61^

In the field of biomarker research, the integration of multi-omics data has successfully identified multiple molecular targets with potential clinical value. Metabolomic analysis identified DPYD and TYMP as key metabolic biomarkers that play important roles in the occurrence, development, and treatment resistance of glioma.^62^ Meanwhile, screening based on plasma proteomics revealed that markers such as ERBB2 and ITGAV are significantly associated with patient overall survival (OS).^63^ More importantly, researchers have discovered through systematic analysis of post-translational modification (PTM) regulatory networks that phosphorylation, acetylation, and other PTMs are highly associated with GBM oncogenic signaling pathways (such as the PI3K/AKT and MAPK signaling pathways). These findings suggest that targeting PTM enzymes (such as kinases or deacetylases) may constitute a novel therapeutic strategy.^64^^,^^65^ These important findings fully demonstrate the significant value of multi-omics analysis in elucidating tumor heterogeneity and guiding personalized treatment.

### **Joint prognostic assessment model of molecular biomarkers and radiomics**

The significant intra-tumoral heterogeneity of GBM limits the predictive efficacy of molecular markers. The integration analysis of molecular features and radiomic features provides more possibilities for the diagnosis and prognosis assessment of GBM.

The detection of IDH mutation status mainly relies on the analysis of biopsy tissue, and biopsy, as an invasive method, has certain limitations in clinical application. Therefore, scholars have proposed establishing an IDH mutation prediction model by combining radiomic features extracted from IDH genotyping with machine learning to construct a radiomics model for predicting IDH mutation status, thereby aiding in prognosis stratification.^66^ The methylation status of the MGMT promoter is a key predictive indicator of chemosensitivity. The construction of an MGMT prediction model using radiomics features from multiparametric MRI can non-invasively assess the methylation level,^38^^,^^67^ which is beneficial for dynamically evaluating the MGMT methylation status in recurrent GBM patients.

GSEA reveals that prognosis-related radiomic features are significantly associated with signaling pathways, immune responses, and other relevant factors,^68–70^ for example, the TRIP6 gene plays a key regulatory role in the NLR pathway, and its expression level can be dynamically associated with radiomic features through single-cell sequencing.^71^^,^^72^ PTEN loss is associated with the activation of specific glial cell transcription pathways (such as ID1 and LAT2), suggesting that it may mediate treatment resistance by regulating metabolic reprogramming.^73^^,^^74^ High expression of the IL7R gene is strongly associated with poor prognosis, and the radiomic model constructed based on this finding is highly predictive in high-grade gliomas.^75^

The combination of traditional molecular biomarkers and novel biomarkers, along with the integration of radiomic models, is fundamentally reshaping the prognosis assessment system for GBM, providing evidence for non-invasive molecular typing. This shift from single-molecular typing to precision prediction driven by multidimensional data is laying a solid foundation for the development of personalized treatment strategies.

### **Machine learning in molecular marker selection**

Machine learning technology has significantly advanced the research progress and clinical application transformation of molecular markers in GBM. In multimodal data integration and key gene screening, machine learning integrates multi-omics data, including genomics, transcriptomics, radiomics, and clinical data, to screen out key molecular markers associated with the malignant progression of GBM. Ensemble learning algorithms, such as random forest algorithms, can effectively identify the expression characteristics of the angiogenesis-related genes CALU, with its expression level is significantly correlated with tumor invasiveness;^76^ feature selection algorithms, such as LASSO regression and support vector machine (SVM) algorithms, are used to explore the important roles of COASY, FTSJ1, and MOGS in the progression and prognosis of lung adenocarcinoma. These methods are also applicable to the systematic mining of GBM molecular markers.^77^^,^^78^ Classification models based on machine learning not only identify GBM prognostic subtypes but also reveal differences in sensitivity to treatment drugs among different subtypes through differential expression analysis. A predictive model constructed using cell senescence-related genes (CSRGs) successfully established a quantitative relationship between gene expression features and patient survival, providing new directions for targeted therapy.^79^

In studies linking radiomics with molecular markers, machine learning models extract texture and morphological radiomic features from MR images, integrating them with glioma molecular characteristics to reveal intrinsic connections between visible images and invisible molecular features. These imaging biomarkers can serve as non-invasive alternatives for molecular typing.^80^ These research findings fully demonstrate the significant role of machine learning in elucidating the heterogeneity of GBM and optimizing diagnostic and therapeutic strategies. However, current research still faces challenges, such as insufficient data standardization and limited model interpretability. Future efforts need to involve multicenter validation and real-time dynamic learning to optimize algorithms further and enhance model reliability.

## Challenges and prospects of molecular markers in clinical practice

### **Standardization and reproducibility issues**

In clinical translational applications, the core challenge of molecular biomarkers lies in standardizing of detection techniques and ensuring data reproducibility. For example, in GBM, there are significant inconsistency in the detection protocols for IDH mutation status, MGMT promoter methylation, and other markers among various research centers. This inconsistency is reflected in multiple aspects, including sample processing procedures, experimental parameter settings, and data analysis methods, which directly affect the comparability of molecular biomarker data across institutions. Owing to the lack of unified detection standards, the critical values of molecular biomarkers are set differently, thereby limiting their application in multicenter studies or clinical guidelines. In the future, it is necessary to develop international consensus and integrate artificial intelligence-assisted analysis to improve the standardization of molecular biomarkers comprehensively.

### **Future research directions and potential breakthroughs**

The significant molecular heterogeneity of GBM requires the development of personalized treatment strategies. The regulation of metabolic-related genes, such as SLC25A22, may reverse radiotherapy resistance,^81^ while PPAR-*γ* agonists can inhibit tumor progression by regulating the ALDOC-PPAR-*γ* axis.^41^ In recent years, the role of epigenetic modifications (such as DNA methylation or histone acetylation) in tumor drug resistance mechanisms has attracted widespread attention, and interventions targeting epigenetic regulatory factors may hope to restore tumor sensitivity to chemotherapy.^82–84^ Additionally, long non-coding RNAs (lncRNAs) are emerging as new research directions for potential diagnostic markers and therapeutic targets.^85^

The high heterogeneity and treatment resistance of GBM have driven the rapid development of multi-omics integration studies. By integrating genomic, transcriptomic, proteomic, metabolomic, and spatial omics data, researchers can systematically analyze tumor cell subpopulations, interactions within the tumor microenvironment, and dynamic evolution patterns. The combined application of single-cell sequencing and spatial transcriptomics further elucidates intra-tumoral heterogeneity, providing new insights for the implementation of precision medicine.^86^^,^^87^ Additionally, the establishment of organoid models and patient-derived xenograft (PDX) models can more realistically simulate tumor microenvironment characteristics, thereby improving drug screening efficiency.^88^^,^^89^ Although multi-omics integration studies provide new perspectives for a deeper understanding of the biological characteristics of GBM and the development of precise treatment strategies, key scientific issues such as the standardization of key technologies, the optimization of data integration algorithms, and the elucidation of dynamic regulatory mechanisms still need to be addressed in the process of clinical translation.

Future research should validate the clinical applicability of existing biomarkers using large-scale samples and explore the synergistic effects of multi-omics integration models in recurrence prediction, aiming to overcome the current clinical challenges in GBM treatment.

## Discussion

In the diagnostic and treatment system of GBM, the mutation status of IDH serves as the cornerstone of GBM molecular typing, owing to the metabolic regulatory functions of different mutation statuses. The metabolic regulation mediated by wild-type IDH leads to poorer survival outcomes and a more complex tumor microenvironment. The methylation of the MGMT promoter can predict chemosensitivity to temozolomide, with methylated patients showing significant benefit from TMZ chemotherapy. Additionally, EGFR amplification, TERT promoter mutations, and chromosomal markers such as +7/-10 not only assist in diagnosis but also reveal tumor heterogeneity, providing evidence for targeted therapy. Emerging biomarkers such as CDKN2A/B deletion and XRN2 further refine prognostic assessment and link to recurrence patterns, collectively advancing personalized medical practice.

Notably, according to the fifth edition of the “Classification of Tumors of the Central Nervous System,” in IDH wild-type high-grade gliomas, H3K27M mutation and H3G34 mutation respectively define diffuse midline glioma, H3K27-altered glioma and diffuse hemispheric glioma, H3G34-mutant, which are no longer classified as IDH wild-type glioblastoma.^90^ The H3K27M mutation is highly enriched in midline structures (such as the pons, thalamus, and spinal cord), making it a core diagnostic criterion for midline gliomas; whereas the H3G34R mutation is specifically localized to the cerebral hemispheres and is associated with non-midline gliomas. In terms of molecular mechanisms, the H3K27M mutation leads to the global destruction of H3K27me3 through dominant-negative PRC2 inhibition, while the H3G34R mutation results in the local loss of H3K36me3 by inhibiting SETD2. Both mutations are associated with poorer survival outcomes.^91^ The H3K27M mutation is often accompanied by higher rates of NF1 and PIK3CA/PIK3R1 mutations, while the H3G34 mutation is more prone to abnormalities in the cell cycle pathway, such as CDK4/CDK6 amplification and CDKN2A/B deletions. This study also provides potential therapeutic targets for immune-targeted treatments.^92^

Molecular biomarkers can guide individualized drug selection for GBM by revealing the specific biological characteristics of tumors. In addition to the well-known methylation of the MGMT promoter, which enhances the drug sensitivity of TMZ, other influences are reflected mainly in aspects such as targeting specific pathways and predicting drug resistance. Tumors with EGFR amplification may be more sensitive to tyrosine kinase inhibitors targeting the pathway, such as Osimertinib, and their mechanism involves the inhibition of the MAPK/ERK pathway; however, these agents need to be used in combination with PI3K/AKT/mTOR pathway inhibitors to exert antitumour activity.^93^ In terms of overcoming drug resistance, glioblastoma cells can mediate TMZ resistance through thioredoxin reductase 1 (TrxR1), while the small molecule inhibitor BS1801 relieves TMZ resistance by targeting TrxR1 and increasing the level of reactive oxygen species.^94^ Additionally, although bevacizumab can improve the progression-free survival of GBM, it requires molecular marker screening to identify the patients who will benefit most. The study revealed that EGFR amplification was more common in patients treated with bevacizumab for  ≤6 months (46% vs. 20%). Multivariate analysis showed that CDK4 amplification may be a potential genetic biomarker for identifying patients who may benefit from long-term bevacizumab treatment.^95^

With the emergence of new molecular biomarkers, research on the tumor microenvironment (TME) and immune checkpoint inhibitors (ICIs) in GBM has increased. Although ICIs have shown efficacy in various types of tumors, clinical trial results in GBM are generally poor, with their ineffectiveness attributed to the immune-suppressive barrier formed by the TME. The TME is a “cold” tumor environment composed of tumor cells, stromal cells, and various immune cells. Tumor cells reprogram T cells into dysfunctional states or promote a tumor state, secrete immunosuppressive factors, and promote tumor immune tolerance, driving the invasive and treatment-resistant nature of tumors and further limiting the efficacy of ICIs. The loss of T-cell antitumor function leading to immune escape is considered one of the key factors in the failure of ICIs treatment.^96^ At the molecular level, immune suppression involves mechanisms such as the activation of inflammasomes mediated by DNA sensors and metabolic pathways of adenosine, while the upregulation of immune checkpoints such as PD-1 also participates in the immune suppression process.^97-99^ To increase the efficacy of ICIs, current research focuses on combination strategies, such as using tumor treatment fields (TTFields) to activate the type I interferon pathway and induce in situ immune sensitization,^99^ or employing nanotechnology for the local delivery of immunomodulators to reverse immune suppression and synergize with anti-PD-1 therapy.^100^ In addition, strategies targeting adenosine metabolism, anti-angiogenesis, and epigenetic regulation are also considered potential directions for reshaping the microenvironment and overcoming immune resistance.^97^^,^^101^ Therefore, an in-depth analysis of the heterogeneity of the GBM microenvironment and developing targeted immune modulation combination therapies are important future directions to enhance the effectiveness of ICIs.

Research on PBZ for glioblastoma also reveals the complexity of its microenvironment and its relationship with tumor recurrence.^44^ However, the regulatory mechanisms of the immune microenvironment within the PBZ and its interactions with glioma stem cells (GSCs) remain unclear. Current evidence indicates that in the PBZ, the proportion of tumor cells and immune cells infiltration is significantly greater in higher brain blood flow regions (HBI) compared to lower brain blood flow regions (LBI).^44^ Therefore, HBI is a potential area for postoperative tumor recurrence, which may be related to the perivascular enrichment of GSCs. Additionally, CD133 is widely regarded as a marker for GSCs and is co-expressed with other molecules (such as CD44 or Nestin), forming a complex signaling network that participates in signal transmission in the microenvironment, promoting the invasive nature and treatment resistance of tumors.^102^ CD133 + GSCs also stabilize the stem cell phenotype and promote tumor initiation through the CD133-Akt-SLC1A5 signaling axis.^103^ These mechanisms are not yet fully clear, but they help explain the recurrence of tumors in the PBZ.

Although research on the PBZ provides certain physiological and pathological bases for surgical margin planning, surgical operations are more reliant on imaging results. Although an expanded resection range has been attempted to improve prognosis, the molecular characteristics of residual tumors at the surgical margins, such as their unique immune microenvironment or drug-resistant signaling pathways, are still in the exploratory stage. This limitation restricts the ability of molecular markers to directly guide the extent of resection.

Owing to the high heterogeneity of GBM, there are relatively fewer studies on how molecular markers affect the different recurrence patterns of GBM, and the specific mechanisms are not yet clear. For example, MGMT promoter methylation has been associated with non-local/distant recurrence in several meta-analyses and cohort studies; EGFR extracellular domain mutations (such as A289 site mutations) are associated with marginally/invasive recurrence; and XRN2 functions in cell migration/invasion, suggesting its possible involvement in distant recurrence/dissemination, but these associations lack sufficient validation from large cohort studies. Most studies have analyzed and discussed the impact of single molecular biomarkers on the prognosis and recurrence of GBM. In the future, we can conduct a correlation analysis of multi-molecular biomarkers with the prognosis and recurrence patterns of GBM. By exploring the impact of the number of molecular biomarker mutations on recurrence patterns and survival, we can indirectly verify whether there is an additive or antagonistic effect between different molecular biomarkers. Moreover, exploring potential molecular biomarkers for predicting GBM recurrence patterns may impact the radiation therapy planning for patients. Although GBM molecular typing has become an important prognostic indicator, the current clinical target volume (CTV) delineation standards (such as expanding the GTV by 1−2 cm) have yet to integrate this molecular information to achieve individualized optimization. Traditional anatomical imaging-defined tumor boundaries may miss microinvasive foci. If specific molecular typing can indicate specific recurrence patterns, clinicians can dynamically adjust the target area or dose during the initial radiation therapy plan, implementing preventive radiation therapy and avoid unnecessary radiation damage to normal tissues. This can significantly reduce the recurrence rate of GBM and the side effects after radiation therapy. Currently, there is still a lack of mature clinical schemes directly guiding radiation therapy target design based on molecular markers. Future research can explore the correlation between GBM recurrence patterns and molecular markers to promote individualized radiation therapy based on tumor biological behavior.

In addition, although existing studies have established prognostic prediction models using metabolic-related gene pairs (MRGPs), glycolysis-related genes (GRGs), or multimodal imaging-molecular data, these models still face issues with standardization and universality in clinical practice. For example, while metabolic characteristics based on 21-MRGP can effectively distinguish high- and low-risk patients, the integration method with the current WHO molecular typing system and its guidance for personalized treatment choices still need to be validated through prospective clinical studies. Recent studies have shown that prognosis models based on glycolysis-related genes have limitations when validated across cancer types, which underscores the importance of studying the specific metabolic‒immune interaction characteristics of IDHwt-GBM. For example, the mechanism by which CLEC5A regulates immune cell infiltration through the PI3K/Akt pathway in IDH wild-type GBM may serve as a potential new target for combined immunotherapy.^7^ Most current research focuses on the analysis of single-omics data, and in the future, it is necessary to explore how to effectively combine metabolic omics characteristics with imaging omics markers to develop decision-support systems that are convenient for clinical use.

Despite the critical importance of biomarkers for the precise diagnosis and treatment of GBM, multiple severe challenges in guiding clinical treatment decisions still exist. The high heterogeneity of GBM makes it difficult for a single biomarker to comprehensively reflect the tumor state, and its dynamic evolution characteristics make static detection unreliable for predicting drug resistance and recurrence. Moreover, the number of biomarkers with clear clinical significance is very limited and is mainly confined to IDH mutations and MGMT mutations. Although many emerging biomarkers have potential, they lack standardized detection methods and large-scale multicenter clinical validation, especially effective biomarkers for predicting immune therapy response and resistance. The value of PD-L1 as an immune therapy biomarker has also not shown significant benefits in clinical trials.

In addition, it is also necessary to address the challenge of converting laboratory research results into clinical applications. There are inherent bottlenecks in obtaining and detecting molecular markers: tissue biopsies have difficulty capturing the spatial and temporal heterogeneity of tumors, while the blood‒brain barrier limits the sensitivity of marker detection in peripheral blood. The clinical utility of alternative sources, such as cerebrospinal fluid, has also not been fully validated. Ultimately, the gap between basic discoveries and clinical applications remains significant because of the interference of tumor heterogeneity with the stability of markers, technical bottlenecks in in vivo delivery (such as CAR-T cells and mitochondrial translation inhibitors), the difficulty of integrating multimodal data, and the lack of collaborative mechanisms between academia, industry, and clinical practicality assessment systems. These validation and standardization challenges urgently need to be addressed through systematic, prospective, multicenter collaborative research.

With respect to the above issues, future research can combine single-cell transcriptomics and spatial metabolomics analysis to reveal the associations between the expression profiles of metabolic genes and spatiotemporal changes in immune cell phenotypes. Additionally, in vitro coculture models can be used to validate key regulatory nodes, such as the impact of ABCA1 inhibitors combined with CAR-T-cell therapy on TAM polarization. Establishing a framework for combined metabolic and immunological analysis and evaluating the synergistic effects of ABCA1 inhibitors and PD-1 blocking agents using preclinical models may provide new insights for improving the immunosuppressive microenvironment. By utilizing patient-derived organoid (PDO) models for drug screening and combining single-cell sequencing to analyze subtype-specific metabolic and epigenetic interaction networks, new targeted therapeutic strategies may be developed. Subsequent studies could elucidate the paracrine interaction networks between specific immune subpopulations in HBI (such as CX3CR1+macrophages) and GSCs (CD133+/SOX2+) by combining single-cell multiomics techniques with organoid co-culture models, and screening for small molecule inhibitors or CAR-T-cell therapies targeting the immunosuppressive microenvironment within the PBZ. Subsequent research should focus on establishing a multicenter, multi-omics joint validation platform and applying artificial intelligence algorithms to improve the interpretability and clinical applicability of the models.

The diagnostic and treatment system for IDHwt-GBM patients is undergoing a major transformation from single biomarker analysis to multidimensional integration. The key for future research lies in establishing a bidirectional connection between molecular mechanisms and clinical translational application, continuously iterating and optimizing through basic research and clinical trials to ultimately drive the innovation of precision diagnosis and therapeutic systems for IDHwt-GBM.

## Conclusion

The molecular heterogeneity of GBM profoundly affects its prognosis and recurrence patterns. This study systematically elucidates the core value of classical molecular markers (IDH mutations, MGMT methylation, EGFR amplification, and TERT mutations) in prognosis stratification. This study reveals the potential of emerging markers (such as XRN2, CRYAB, and immune metabolic regulators) through interventions in metabolic reprogramming, invasion and metastasis, and the immune microenvironment, providing new directions for targeted therapy. Recurrence studies have confirmed that the spatial molecular characteristics of PBZ (enrichment of the mesenchymal phenotype, co-expression of CRYAB and CD44) are closely related to anatomical preference recurrence. Meanwhile, the predictive potential of EGFR mutations and the MGMT methylation status for recurrence patterns may optimize individualized radiotherapy strategies.

Current liquid biopsies, multi-omics integration, and joint models combining imaging and molecular approaches are driving innovations in non-invasive, dynamic, and precise diagnosis and treatment. However, they still face challenges such as insufficient standardization of detection, interference from spatial heterogeneity, and the complexity of technological integration. In the future, through multicenter validation, artificial intelligence-driven learning, and cross-platform research, it will be necessary to deepen our understanding of the metabolic-immune interaction mechanisms in IDHwt-GBM, the dynamic evolution of treatment resistance, and the regulatory network of the PBZ microenvironment. Ultimately, this will achieve a paradigm shift from single markers to multi-dimensional integration, laying the foundation for an individualized, precise medical treatment system.

## Data Availability

Data sharing is not applicable to this article, as no new data were created or analyzed in this study.
